# Robustness of muscle synergies during visuomotor adaptation

**DOI:** 10.3389/fncom.2013.00120

**Published:** 2013-09-03

**Authors:** Reinhard Gentner, Timothy Edmunds, Dinesh K. Pai, Andrea d'Avella

**Affiliations:** ^1^Laboratory of Neuromotor Physiology, Santa Lucia FoundationRome, Italy; ^2^Department of Computer Science, University of British ColumbiaVancouver, BC, Canada

**Keywords:** muscle synergies, visuomotor rotation, motor adaptation, isometric force, EMG, directional tuning

## Abstract

During visuomotor adaptation a novel mapping between visual targets and motor commands is gradually acquired. How muscle activation patterns are affected by this process is an open question. We tested whether the structure of muscle synergies is preserved during adaptation to a visuomotor rotation. Eight subjects applied targeted isometric forces on a handle instrumented with a force transducer while electromyographic (EMG) activity was recorded from 13 shoulder and elbow muscles. The recorded forces were mapped into horizontal displacements of a virtual sphere with simulated mass, elasticity, and damping. The task consisted of moving the sphere to a target at one of eight equally spaced directions. Subjects performed three baseline blocks of 32 trials, followed by six blocks with a 45° CW rotation applied to the planar force, and finally three wash-out blocks without the perturbation. The sphere position at 100 ms after movement onset revealed significant directional error at the beginning of the rotation, a gradual learning in subsequent blocks, and aftereffects at the beginning of the wash-out. The change in initial force direction was closely related to the change in directional tuning of the initial EMG activity of most muscles. Throughout the experiment muscle synergies extracted using a non-negative matrix factorization algorithm from the muscle patterns recorded during the baseline blocks could reconstruct the muscle patterns of all other blocks with an accuracy significantly higher than chance indicating structural robustness. In addition, the synergies extracted from individual blocks remained similar to the baseline synergies throughout the experiment. Thus synergy structure is robust during visuomotor adaptation suggesting that changes in muscle patterns are obtained by rotating the directional tuning of the synergy recruitment.

## Introduction

Human subjects can learn to move in novel environments and they can adapt to visuomotor (Ghilardi et al., 1995; Imamizu et al., 1995; Ghahramani et al., 1996; Krakauer et al., 1999, 2000) or dynamic (Lackner and Dizio, 1994; Shadmehr and Mussa-Ivaldi, 1994) perturbations. Generally, when subjects are exposed to a perturbation of the mapping between motor commands and end-effector motion or force, they initially produce large errors and they then gradually adapt, compensating for the perturbation and re-establishing baseline performance. When the perturbation is removed subjects make large errors in the opposite direction (after-effects) before gradually re-adapting. If the perturbation is unexpectedly and occasionally removed in a single trial (Thoroughman and Shadmehr, 2000) or if it changes continuously and randomly (Scheidt et al., 2001; Baddeley et al., 2003; Cheng and Sabes, 2007) the error experienced in one trial affects the motor command generated in the following trial. These observations suggest that the central nervous system (CNS) relies on internal models of the body and of the environment to predict the sensory consequences of motor commands and that adaptive processes adjust the internal models to reduce sensory prediction errors (Shadmehr et al., 2010; Krakauer and Mazzoni, 2011; Wolpert et al., 2011). Such adaptive processes can be modeled as error-based learning that reduces sensory prediction error by adjusting an internal state according to a linear time-invariant dynamics (Thoroughman and Shadmehr, 2000; Donchin et al., 2003; Cheng and Sabes, 2007; Tanaka et al., 2012). Multiple learning processes operating at different timescales (Smith et al., 2006) and learning at different hierarchical levels in the internal model (Braun et al., 2009) explain the time-course of performance errors under a variety of experimental manipulations. However, albeit behavioral observations, such as error time-course and generalization properties, made in numerous motor adaptation studies are well captured by current models, how the motor commands change during motor adaptation has been investigated only in a few cases (Wise et al., 1998; Thoroughman and Shadmehr, 1999; Li et al., 2001; Paz et al., 2003; de Rugy et al., 2009). Muscle pattern generation and its relationship to force generation during motor adaptation still needs to be fully understood.

Because of the redundancy in the musculoskeletal system, the change in motor commands underlying the change in motion or force necessary to compensate for a visuomotor or dynamic perturbation is not unique. For example, the rotation of the direction of force required to adapt to a rotation imposed onto the mapping between the force applied on an isometric joystick and the motion of a cursor on a computer screen (visuomotor rotation) may be accomplished by infinitely many different combinations of changes in individual muscle activations. In principle, during motor adaptation the performance error may be gradually reduced by changing the force output associated to each visual target using the same muscle pattern used for that force output before the perturbation. Alternatively error may be reduced by changing the activity of individual muscles independently of the muscle patterns used before the perturbation. For wrist muscles it has been shown that the rotation of the muscle directional tuning curve closely follows the rotation imposed onto the force-to-cursor mapping (de Rugy and Carroll, 2010), suggesting that adaptation occurs at the level of the planned force output. The first aim of our study was to investigate if this is also true for shoulder and elbow muscles during visuomotor rotation of the mapping between isometric forces generated by the arm at the hand, i.e., with a musculoskeletal system involving a larger number of muscles and joints.

The changes of the motor commands underlying adaptation to a visuomotor rotation may occur at the target force or at the muscle level, but in both cases the question of how a specific muscle pattern is selected to generate a desired force remains open. One hypothesis which has recently received considerable attention is that muscle patterns are generated as combinations of a few muscle synergies, coordinated recruitment of groups of muscles with specific activation balances, thus requiring the selection of only a small number of synergy combination parameters to generate a desired force. While muscle synergies have been studied intensively in human reaching movements (d'Avella et al., 2006, 2008, 2011; Muceli et al., 2010), isometric force generation (Borzelli et al., 2012; Roh et al., 2012), locomotion (Ivanenko et al., 2004; Dominici et al., 2011), cycling (Hug et al., 2010, 2011), responses to postural perturbations (Krishnamoorthy et al., 2003; Torres-Oviedo and Ting, 2007; Chvatal and Ting, 2012), complex motor skills (Frere and Hug, 2012), and in several different animal behaviors (Tresch et al., 1999; Saltiel et al., 2001; d'Avella et al., 2003; Hart and Giszter, 2004; Cheung et al., 2005, 2009; d'Avella and Bizzi, 2005; Ting and Macpherson, 2005; Torres-Oviedo and Ting, 2007; Overduin et al., 2008, 2012; Hart and Giszter, 2010), muscle synergies have not been directly investigated during adaptation to visuomotor rotations. Thus, our second aim was to investigate whether the synergies capturing the muscle patterns underlying the generation of multidirectional isometric forces are robust during motor adaptation. Thus, we hypothesized that the change in the tuning of muscles during adaptation to visuomotor rotation closely follows the rotation of the force and that the underlying changes in the muscle patterns can be explained by changes in the recruitment of synergies whose structure remains fixed.

## Materials and methods

### Participants

All procedures were approved by the Ethical Review Board of Santa Lucia Foundation. Eight right-handed naïve subjects (mean age 28.6 ± 6.0 year, age range 24–43, 5 females and 3 males, see Table 1) participated in the experiments after giving written informed consent.

**Table 1:** **Summary of characteristics and results for individual participants**.

**Subject**	**Age**	**Sex**	**Number of tuned muscles**	***R*^2^ of cosine fit mean ± SD (range)**	**Number of synergies**	**Number of tuned synergies**	***R*^2^ of cosine fit mean ± SD (range)**
1	24	Male	8	0.76 ± 0.16	4	2	0.74 ± 0.15
				(0.49–0.96)			(0.56–0.91)
2	26	Female	9	0.75 ± 0.17	5	3	0.73 ± 0.14
				(0.46–0.92)			(0.54–0.86)
3	43	Male	9	0.78 ± 0.19	4	4	0.88 ± 0.02
				(0.32–0.95)			(0.86–0.90)
4	28	Female	8	0.75 ± 0.17	5	3	0.74 ± 0.11
				(0.41–0.92)			(0.63–0.91)
5	28	Male	8	0.73 ± 0.24	4	3	0.82 ± 0.10
				(0.02–0.98)			(0.68–0.90)
6	25	Female	8	0.68 ± 0.23	4	4	0.83 ± 0.05
				(0.18–0.88)			(0.78–0.88)
7	27	Female	9	0.73 ± 0.19	5	3	0.64 ± 0.33
				(0.25–0.95)			(0.12–0.94)
8	28	Female	9	0.72 ± 0.26	5	3	0.74 ± 0.23
				(0.07–0.94)			(0.47–0.93)
Mean	28.6		8.5	0.74 ± 0.20	4.5	3.1	0.77 ± 0.14
				(0.28–0.94)			(0.58–0.91)

### Experimental setup

**Figure 1:**
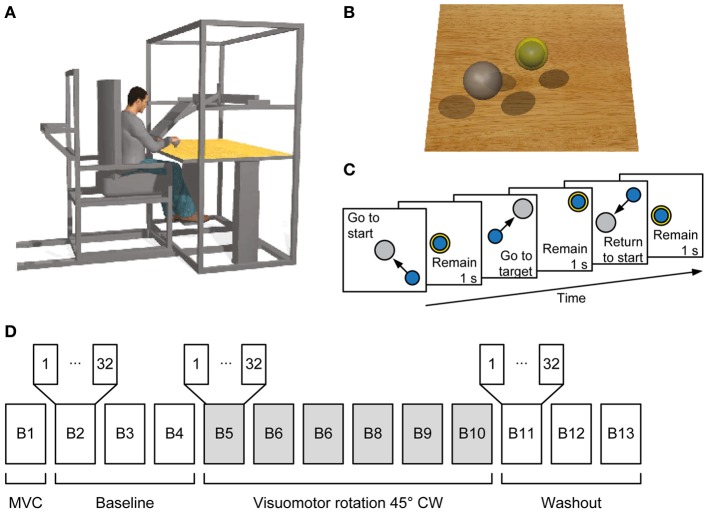
**Experimental setup and procedures. (A)** Subjects sat in a moveable chair with their forearm pronated and fixed in a splint rigidly coupled to a force transducer. A flat monitor occluded the subject's hand and displayed a virtual scene co-located with the real desktop. **(B)** Screenshot of the virtual scene. Subjects controlled the position of the blue sphere by applying forces to the force transducer. The sphere is illustrated inside the yellow semi-opaque sphere indicating the start position. The target is shown as a gray sphere. **(C)** Sequence of events in a trial. Subjects had to maintain the blue sphere inside the start sphere for 1 s. Afterwards the target appeared and the start sphere disappeared instructing the subject to reach it and to hold the blue sphere inside the target sphere for 1 s. The target sphere changed its color from gray to yellow when the target was reached. Finally, the subject was instructed to return to the start position and remain there for 1 s. **(D)** Organization of an experimental session. In the first block the maximum voluntary contraction (MVC) during generation of maximum voluntary force across directions was established followed by three baseline blocks, each consisting of 32 trials. From the fifth block to the tenth block a clockwise (CW) visuomotor rotation was introduced followed by three washout blocks without visuomotor rotation.

Subjects sat in front of a desktop with their torso immobilized by safety belts. Their right forearm was inserted into a splint immobilizing the hand, wrist, and forearm. The center of the palm was aligned with the body midline at the height of the sternum and the elbow was flexed approximately by 90°. The subjects' view of the hand was occluded by a 21-inch LCD monitor inclined with its surface approximately perpendicular to the subjects' line of sight when looking at their hand (Figure 1A). After a calibration procedure, the monitor could display a virtual desktop matching the real desktop, a spherical cursor matching, at rest, the position of the center of the palm and moving on a horizontal plane, and spherical targets on the same plane (Figure 1B). A steel bar at the base of the splint was attached to a 6-axis force transducer (Delta F/T Sensor, ATI Industrial Automation, Apex, NC, USA) positioned below the desktop to record isometric forces. Surface electromyographic (EMG) activity from 13 muscles acting on the shoulder and elbow muscles was recorded with active bipolar electrodes (DE 2.1, Delsys Inc., Boston, MA), after band-pass filtering (20–450 Hz) and amplification (gain 1000, Bagnoli-16, Delsys Inc.). Force and EMG data were digitized at 1 kHz using an A/D PCI board (PCI-6229, National Instruments, Austin, TX, USA). The virtual scene was rendered by a PC workstation with a refresh rate of 60 Hz using custom software. Cursor position information was processed by a second PC workstation running a real-time operating system and transmitted to the first workstation through an Ethernet link. Cursor motion was simulated in real time as a mass accelerated by the horizontal force (parallel to the desktop) applied by the subject on the splint, a viscous force, and an elastic force proportional to the distance for the rest position. The spring constant was set such that a constant force with a magnitude equal to 20% of the mean maximum voluntary force (MVF) magnitude across force directions (see below) would maintain the cursor stationary at 5 cm from the origin. The damping constant was set to make the system critically damped.

### Experimental protocol

The experiment was subdivided into blocks, each consisting of a set of trials (Figures 1C,D). The first maximum voluntary contraction (MVC) block served to establish a mean MVF over horizontal force directions of each subject. At each trial subjects moved the sphere along a virtual line in one of 8 directions (equally spaced by 45°) by applying horizontal forces until they reached their maximum force production capability. After remaining 1 s at the position of maximum force, subjects were instructed to relax and to bring back the sphere to the rest position. When the trial stopped after 15 s, a new trial with a different target direction was initiated. In the following blocks, subjects performed center out forces to 8 equally spaced targets with force levels of 20 and 30% of MVF (corresponding to displacements of 5 and 7.5 cm of the sphere, respectively). Each target was repeated two times in a pseudorandom order (i.e., 32 trials per block). A trial was initiated by keeping the sphere at the start position (tolerance ±2% of MVF, i.e., 0.5 cm) for 1 s. Afterwards, a target appeared and the sphere indicating the start position disappeared. Subjects were instructed to move to the target as fast as possible, and to remain for 1 s at the target (tolerance ±2% of MVF). The trial was finished successfully 0.5 s after returning to the start position (Figure 1C).

Subjects performed three blocks of 32 trials (baseline), followed by six blocks with a 45° clockwise (CW) visuomotor rotation applied to the planar force used to compute the cursor displacement (rotation), and another three blocks without the rotation (washout) as shown in Figure 1D.

### Data analysis

#### Initial directional error

**Figure 2:**
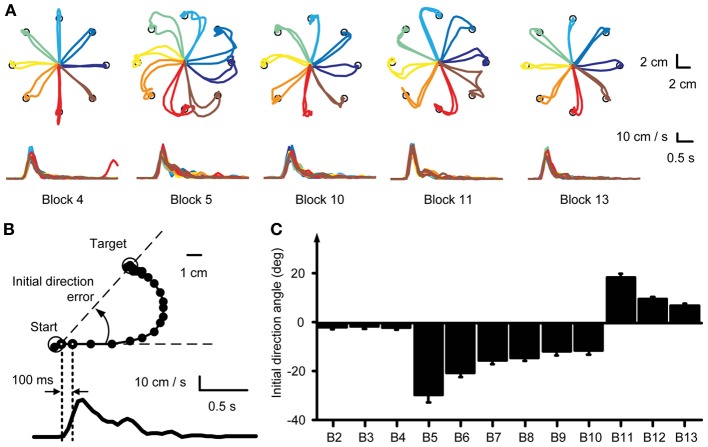
**Kinematic analysis. (A)** Example force traces to targets located at a distance 30% of MVF. Trajectories to the targets were straight in the baseline block (Block 4) and curved at the beginning of the visuomotor perturbation (Block 5). With practice subjects adapted to the rotation and trajectories were again straight at the end of the exposure to the perturbation (Block 10). Aftereffects were present at the beginning of the washout (Block 11). At the end of the washout (Block 13) subjects re-adapted and were again able to produce straight trajectories to the target. **(B)** The initial movement direction angle with respect to a straight line to the target established at 100 ms after movement onset was used as a measure to quantify the adaptation process. **(C)** Mean initial direction error across subjects for each block.

To evaluate the adaptation to the visuomotor rotation at the kinematics level we computed the initial directional error at 100 ms after movement onset. Movement onset was defined when the cursor speed exceeded 0.5 cm s^−1^. The initial directional error was defined as the angle of the vector pointing from movement onset to the cursor position at 100 ms after movement onset with respect to a straight line to the target (Figure 2B).

#### Synergy extraction

Muscle synergies from each block were identified by non-negative matrix factorization (NMF) from EMG patterns recorded from the go signal to the end of successful target acquisition. Recorded EMG data were rectified and digitally low-pass filtered (2nd order Butterworth, 5 Hz cutoff) and re-sampled at 100 Hz to reduce data size. In each trial, mean EMG activity of each muscle during the initial rest phase was used as an estimate of baseline noise level and subtracted from the rest of the data. The EMGs were normalized to the maximum activation across direction recorded during the MVC block. Finally, the rectified and normalized EMGs of each trial from a given block (or from several blocks) were pooled together into a single data matrix **M**. The concatenated EMG patterns **m** (columns of the matrix **M**) were described by a combination of synergy coefficient by **m** = **W c**, with **W** the *M* × *N* synergy matrix whose columns are vectors specifying relative muscle activation levels (invariant across time and trials), and **c** a *N*-dimensional synergy activation vector (time- and trial-dependent), *N* the number of synergies and *M* the number of muscles. The number of data points (columns) in the matrix **M** slightly varied between blocks and subjects because the time to successfully complete the target acquisition was not constant for each trial. For each possible *N* from 1 to *M*, the iterative optimization algorithm (Lee and Seung, 1999, 2001) was repeated 10 times and the solution with the highest fraction of data variation explained (*R*^2^) was retained. We selected the smallest number of synergies which explained more than 90% of the data variation. Synergies were extracted from the following 13 muscles: Brachioradialis (BracRad), Biceps brachii, short head (BicShort), Biceps brachii, long head (BicLong), Triceps brachii, lateral head (TrLat), Triceps brachii, long head (TrLong), anterior Deltoid (DeltA), medial Deltoid (DeltM), posterior Deltoid, posterior (DeltP), clavicular part of the Pectoralis major (PectMajClav), medial Trapezius (TrapMed), Latissimus dorsi (LatDorsi), Teres Major (TerMaj) and Infraspinatus (InfraSp).

#### Tuning curves

**Figure 3:**
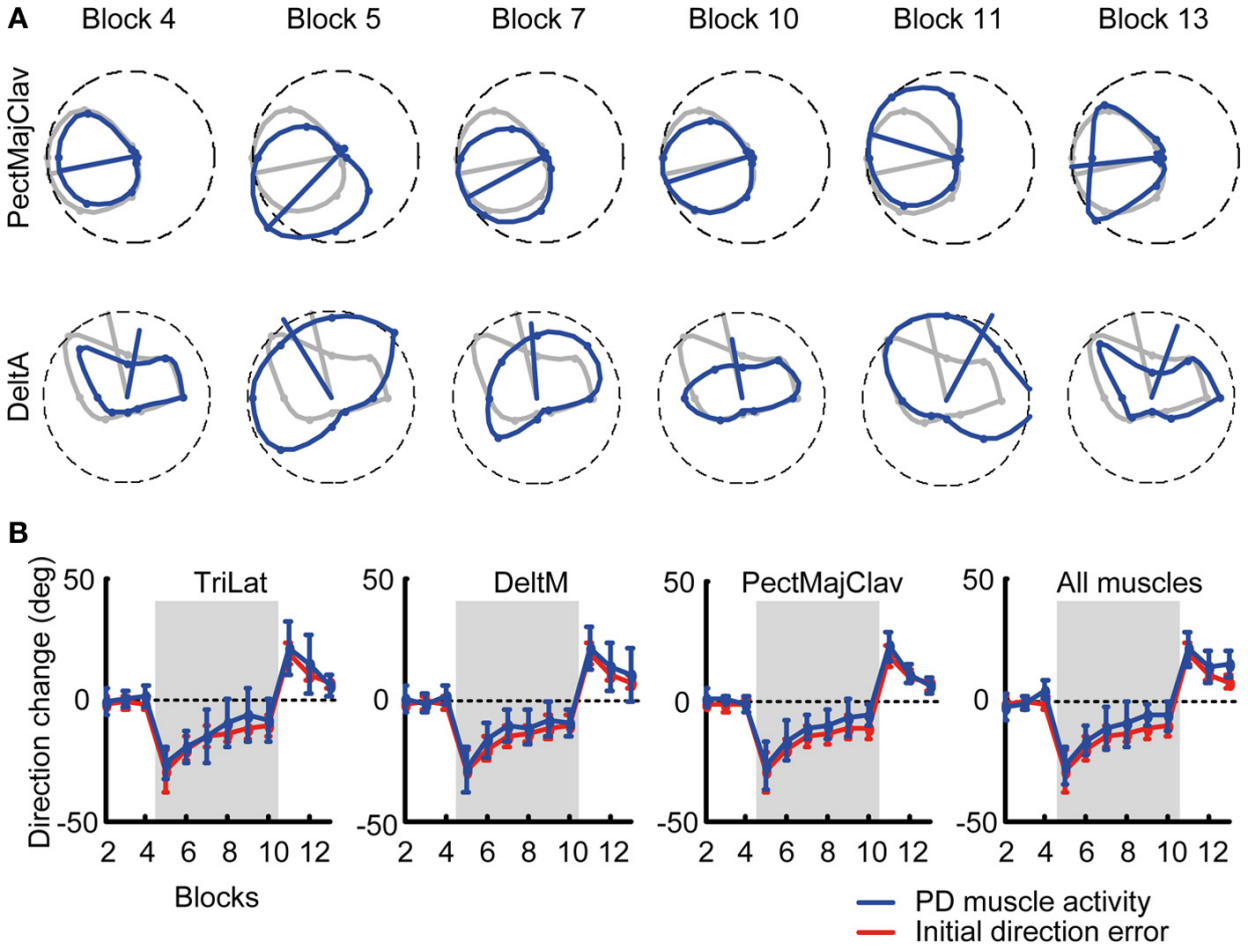
**Tuning curves and preferred directions of individual muscles. (A)** Example of tuning curves for two muscles (*first row*: PectMajClav, *second row*: DeltA) of subject 3 estimated from different blocks (*blue* markers and lines) compared to the tuning curve of the muscles calculated from the pooled data of all baseline blocks (reference blocks, *gray* markers and lines). As for PectMajClav, the changes in preferred direction (PD, *blue* radial segment) of many muscles with respect to the PD in the reference blocks were similar to the initial direction angle errors. However, the activation of some muscles such as DeltA was not cosine tuned and those muscles were excluded from analysis. **(B)** Examples of the mean PD-change of three muscles across subjects and grand mean of the PD-change across muscles and subjects (*rightmost panel*). The PD changes of the muscles (*blue*) was not statistically different from the initial direction angle error (*red*).

Muscle and synergy tuning curves and preferred directions (PDs) were calculated for each block by a cosine fit (d'Avella et al., 2006) between the activation of each muscle or the coefficients of each synergy (averaged across target distances and repetitions in a block) and the corresponding target position. We fitted the muscle (or synergy) activity with a linear regression *m*(θ) = β_0_ + β_*x*_ cos(θ) + β_*y*_ sin(θ), where *m*(ϑ) is the muscle (synergy) activity for a target in direction ϑ and θ^*PD*^ = tan^−1^ (β_*y*_/β_*x*_) is the PD of the cosine tuning. Tuning curves were computed for the time interval between movement onset and the following 100 ms, equivalent to the computation of the initial movement angle error. For visualization, the tuning curves were smoothed by a 2-dimensional spline interpolation and plotted in a polar coordinate system. Muscles or synergy coefficients which were not significantly cosine tuned were excluded from analysis (see Table 1). Significant cosine tuning was assumed when the *p*-value of the regression between the data and the optimal cosine tuning was smaller than 0.05 (see Table 1 for *R*^2^ values of the regression for each subject). After applying a CW visuomotor rotation, the cursor movement was initially directed CW with respect to the target, i.e., with the same directional error that would have been obtained with a rotation of the target in a counter-clockwise (CCW) direction instead of the CW cursor rotation. Thus, the PDs of muscles and synergies were computed according to the CCW-rotated visual targets, i.e., the actual force targets. Then the initial change of PD is directed CCW as displayed for individual muscles in Figure 3A. To better compare the changes of muscle- and synergy-PDs with the cursor initial direction error, we changed the sign of those PDs in block 5–10.

#### Data reconstruction by synergies

To quantify how well the structure of the muscle patterns of one block were captured by the synergies extracted from different blocks we reconstructed the EMG traces by finding the synergy coefficients that reconstructed those traces with the highest *R*^2^ value. To find the optimal synergy coefficients we ran the same iterative optimization algorithm used for the extraction of the synergies without updating the synergies. We calculated the *R*^2^ value of the reconstruction of EMG traces from blocks 2–13 using the synergies extracted from the pooled data from blocks 2–4. Monte-Carlo simulations were used to ensure that the reconstruction quality was higher than chance level. For the Monte-Carlo simulation we selected 30 sets of EMG signals at randomly chosen time points which reflected sets of *M* × *N* “random” synergies. The number of random synergies for each set and subject was chosen equal to the number of selected synergies. For each set of random synergies the EMG traces were reconstructed and the *R*^2^ value computed.

#### Synergy similarity

We measured the similarity between two synergies by normalizing the synergy vectors (Euclidean norm) and computing their scalar product. Similarity between two sets of synergies was assessed by first matching pairs of synergies according to their normalized scalar product (starting from the pair with the highest value and continuing with the pairs from the remaining synergies until all pairs were matched) and then computing the mean scalar product over all matched pairs. We selected the number of synergies from the reference synergies for all comparisons of different sets of synergies for each subject. As for the reconstruction *R*^2^, Monte-Carlo simulations were used to ensure that the similarity was higher than chance level.

#### Statistical analysis

Two-Way repeated measures ANOVAs (2 adaptation measures × 6 perturbation blocks) were conducted to detect significant differences between the time course of initial direction error and that of change of muscle PDs. Paired *t*-tests with Bonferroni correction were applied where appropriate.

## Results

### Initial direction error changes during visuomotor rotation

In the baseline blocks subjects produced relatively straight trajectories (Figure 2A, Block 4) with bell shaped velocity profiles (Figure 2A, *bottom row*). Change of visuomotor mapping caused distorted trajectories (Figure 2A, Block 5) and multi-peaked velocity profiles reflecting corrective movements necessary to reach the target. After exposure of six blocks with visuomotor rotation subjects compensated (Figure 2A, Block 7) for the perturbation and were able to produce relatively straight movements to the target (Figure 2A, Block 10). Velocity profiles approached a single bell-shaped velocity profile again. When the visuomotor rotation was removed (Figure 2A, Block 11), subjects showed aftereffects in the opposite direction of the perturbation, which were extinguished after three washout blocks (Figure 2A, Block 13).

We quantified the adaptation of all subjects by analyzing the initial movement direction error with respect to a straight line to the target at 100 ms after movement onset (Figure 2B, *right dotted vertical line*). Movement onset was defined as the time when the cursor speed exceeded a threshold of 5 cm/s (Figure 2B, *left dotted vertical line*) after the Go-signal had occurred. Across subjects, the initial movement direction showed a large CW deviation when the rotation was introduced (Block 5, Figure 2C) that was gradually reduced with practice (Blocks 5–10) and a large CCW deviation (aftereffect) once the perturbation was removed (Block 11). One-Way repeated measures ANOVA confirmed a significant difference of initial direction error (factor: block number, *F* = 5.04, *p* < 0.001). In the baseline blocks the initial direction error was small (Block 2: −1.86 ± 2.41°, mean ± SD, Block 3: −1.44 ± 3.05°; Block 4: −1.96 ± 2.64°). At the beginning of the visuomotor perturbation the initial direction error (Block 5: −29.59 ± 8.42°) was significantly different from the error in the last baseline block (*p* < 0.001, two-tailed, paired *t*-test) but approached baseline level by a gradual adaptation in subsequent blocks (Block 10: −11.35 ± 4.53°, *p* = 0.002 with respect to Block 4). After the perturbation was removed, the initial direction error was significantly (Block 11: 18.62 ± 4.33°, *p* < 0.001) higher than in the last baseline block, but approached baseline level at the end of the washout (Block 13: 7.02 ± 2.71°, *p* < 0.001 with respect to Block 4). All comparisons remained significant after Bonferroni correction.

### Preferred direction change of muscles matches initial direction error change

We tested if the PD of muscle directional tuning followed the change of initial direction error, as was observed in visuomotor rotation of isometric wrist movements (de Rugy, 2010). We found that not all recorded muscles were cosine-tuned. Some muscles showed peaks of activity in multiple directions and their tuning was not captured by a single cosine function. We therefore excluded all muscles which were not significantly cosine-tuned in the reference baseline blocks (see Table 1 for *R*^2^ values of the cosine fit) for this analysis as their PDs did not characterize their directional tuning reliably.

Figure 3A shows an example of a typical change of the PD of a cosine-tuned muscle (*blue tuning curves*, PectMajClav) with respect to the PD of the reference blocks (*gray*, Blocks 2–4). Several muscles were found not to be cosine-tuned, for example DeltA shown in Figure 3A (*second row*). The time-course of the PD of DeltA across blocks would indicate a larger PD change in the last baseline block (Block 4) than in the first visuomotor rotation block (Block 5) with respect to the reference blocks. Considering only cosine-tuned muscles the PDs closely followed the change in force direction error as shown for three muscles and the overall mean across muscles and subjects, respectively, in Figure 3B. A Two-Way repeated measures ANOVA comparing the mean change of the muscle PDs across subjects and muscles with the mean initial direction error did neither reveal a difference (adaptation measure × perturbation block, *F* = 0.73, *p* = 0.394) between the two measures nor a significant interaction (*F* = 0.34, *p* = 0.889).

### Robustness of synergy structure

**Figure 4:**
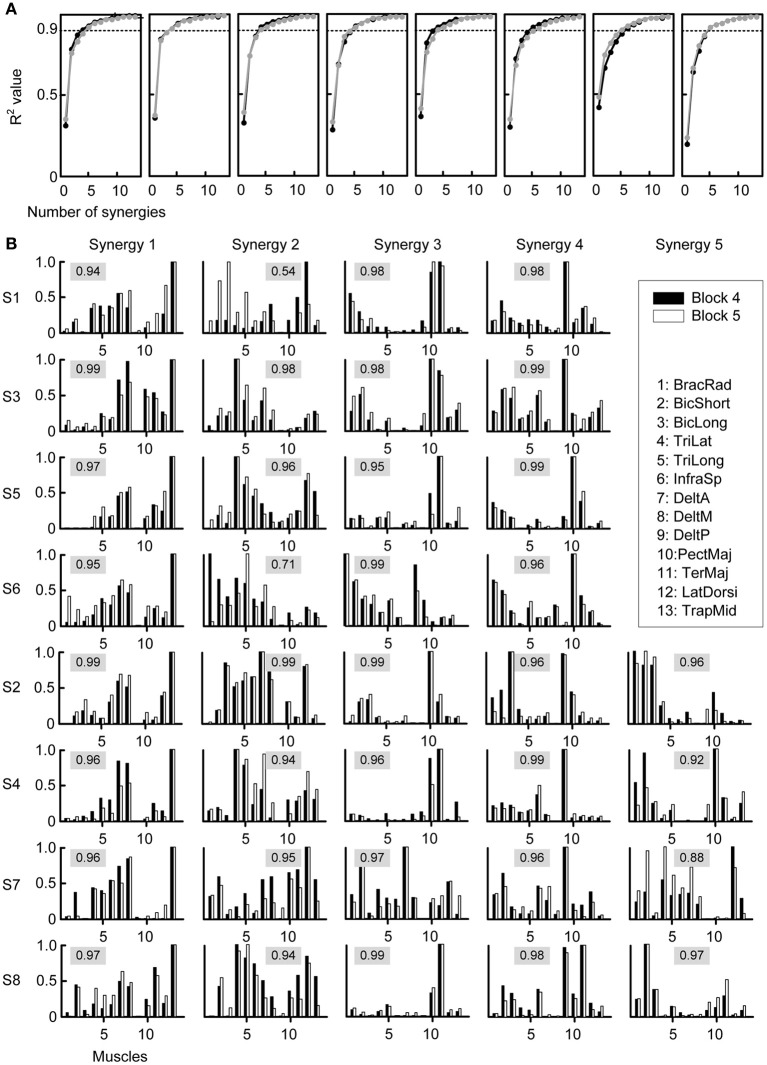
**Selection of number of synergies and synergy structure. (A)** Fraction of total variation explained (*R*^2^) by the synergies extracted from Block 4 (last baseline block, *black*) and by the synergies extracted from Block 5 (first perturbation block, *gray*) were similar for all subjects (*different plots*). **(B)** Synergies from all subjects (*rows*) extracted from Block 4 (*black bars*) and Block 5 (*gray bars*). The numeric value in each plot represents the similarity between the two synergies, quantified by the scalar product of the normalized synergies (cosine-value).

Given the close relationship between muscles and forces we tested whether the adaptation process could be explained by fixed muscle synergies being recruited with PDs rotating together with the muscle PDs. We extracted synergies from each block from the EMG signals beginning at movement onset until the time point at which the target was successfully acquired. Figure 4A shows the fraction of data variation explained by the extracted synergies for the last baseline block (Block 4, *black*) and the first perturbation block (Block 5, *gray*). The number of synergies selected according to a 90% threshold was not significantly (*p* = 0.598, two-tailed paired *t*-test) different between the two blocks (Block 4: 4.75 ± 0.71 synergies, Block 5: 4.62 ± 0.52 synergies). When considering all blocks, the minimum number of synergies explaining more than 90% of the data variation was not significantly different over time, as revealed by a One-Way repeated measures ANOVA (factor: block number, *F* = 1.68, *p* = 0.093).

The structure of the synergies in most cases was similar across subjects and blocks. Figure 4B compares the synergies extracted from Block 4 (black bars) with those extracted from Block 5 (white bars) for each subject. We compared a number of synergies in Block 4 and 5 equal to the number of synergies extracted from the pooled data of Blocks 2, 3, and 4 (“reference synergies,” see Table 1). We identified the best matching pairs of synergies according to the similarity quantified by the cosine between the synergy vectors and plotted them side-by-side (Figure 4B, cosine values are shown with a gray shaded background). In general, the similarity of synergies from Block 4 and 5 across subjects, as indicated by a cosine-value close to one, was high (mean ± SD of similarity: 0.95 ± 0.06, range: 0.54–0.99). However, 2 out of 36 pairs had a similarity <0.8 (S1: second pair, similarity 0.54; S6: second pair, similarity 0.71) and an additional pair had a similarity <0.9 (S7, fifth pair, similarity 0.88). Additional analysis of the synergies in such pairs indicated that they were similar either to one of the synergies in the reference set with the same number of synergies (S6: similarity 0.93 and 0.90 for the synergies extracted from Block 4 and 5 respectively; S1: similarity 0.98 for the synergy extracted from Block 5; S7: similarity 0.93 for the synergy extracted from Block 5) or to one of the synergies in the reference set with an additional synergy (S1: similarity 0.95 for the synergy extracted from Block 4). In one case (S7), the sets of 4 synergies extracted from Blocks 4 and 5 had a much higher similarity (mean 0.97, minimum 0.94) than the sets with the same number of synergies as the reference synergies (5 for S7). These observations suggest that the identification of the synergies from individual blocks is affected by noise and inter-trial variability more than the identification of synergies from the pulled data of all three baseline blocks. Moreover, even if the data of a specific block were best captured by a synergy that did not match closely any synergies in a different block, such synergy might only have captured a very small amount of variation in the data.

**Figure 5:**
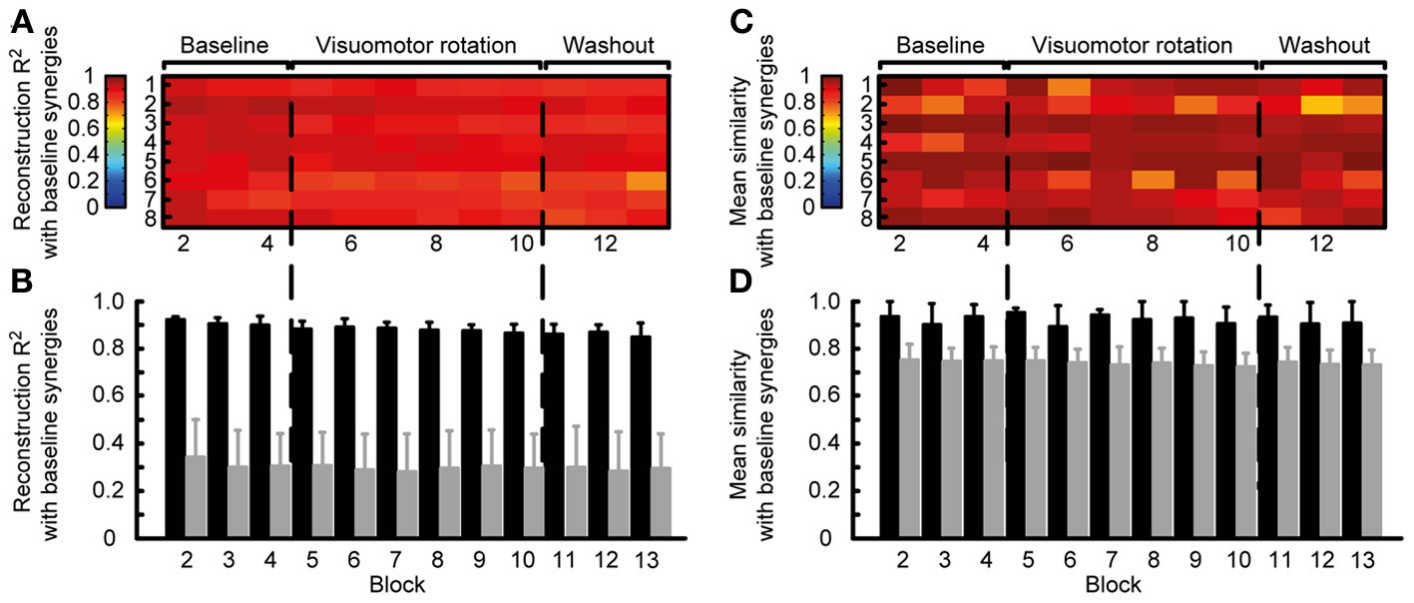
**Robustness of synergies during adaptation. (A)** Quality of the reconstruction of EMGs recorded from different blocks throughout the experiment (*columns*) for each subject (*rows*) by synergies extracted from the reference blocks. **(B)** Mean reconstruction *R*^2^ for each block over subject (*black bars*) compared with the mean reconstruction *R*^2^ for random synergies (*gray bars*). **(C)** Similarity (mean normalized scalar product over best matching pairs) between the synergies extracted from the reference blocks and those extracted from each individual block. **(D)** Mean similarity for each block over subjects (*black bars*) compared with the mean similarity for random synergies.

Thus, we quantified the stability of the subspace spanned by the reference synergies by assessing how well they could reconstruct the muscle patterns of all other blocks. The similarity of the reconstructed muscle patterns (obtained by multiplying synergies extracted from reference blocks and synergy coefficients fitted onto the data of each block) with respect to the actual muscle patterns of each block was quantified as a *R*^2^ value. The high *R*^2^ values (range: 0.72-0.94, mean ± SD: 0.88 ± 0.04) indicate that muscle patterns during adaptation to visuomotor rotation are selected from a stable muscle subspace (Figure 5A). However, there was a small but constant decrease of *R*^2^ values during the experiment, possibly reflecting small changes in elbow position or fatigue.

To exclude that the high *R*^2^ values were obtained by chance, we attempted to reconstruct the data using random synergies. We repeated the reconstruction with 30 sets of random synergies. In all blocks the *R*^2^ value obtained with random synergies was significantly smaller (all *p* < 0.001, two-tailed paired *t*-tests, *gray bars* in Figure 5B) than the reconstruction using synergies extracted from the reference blocks (Figure 5B, *black bars*).

We also assessed the similarity of the synergies extracted from the reference blocks and those extracted from all blocks. In all subjects and blocks, the mean normalized scalar product between the best matched pairs of synergies was close to one (Figure 5C), indicating a high similarity. Across subjects, in all block the similarity (Figure 5D *black bars*, range: 0.69–0.99, mean ± SD: 0.92 ± 0.07) was significantly higher that between random synergies (*gray bars*, *p* < 0.01).

### Adaptation by rotation of synergy preferred direction

**Figure 6:**
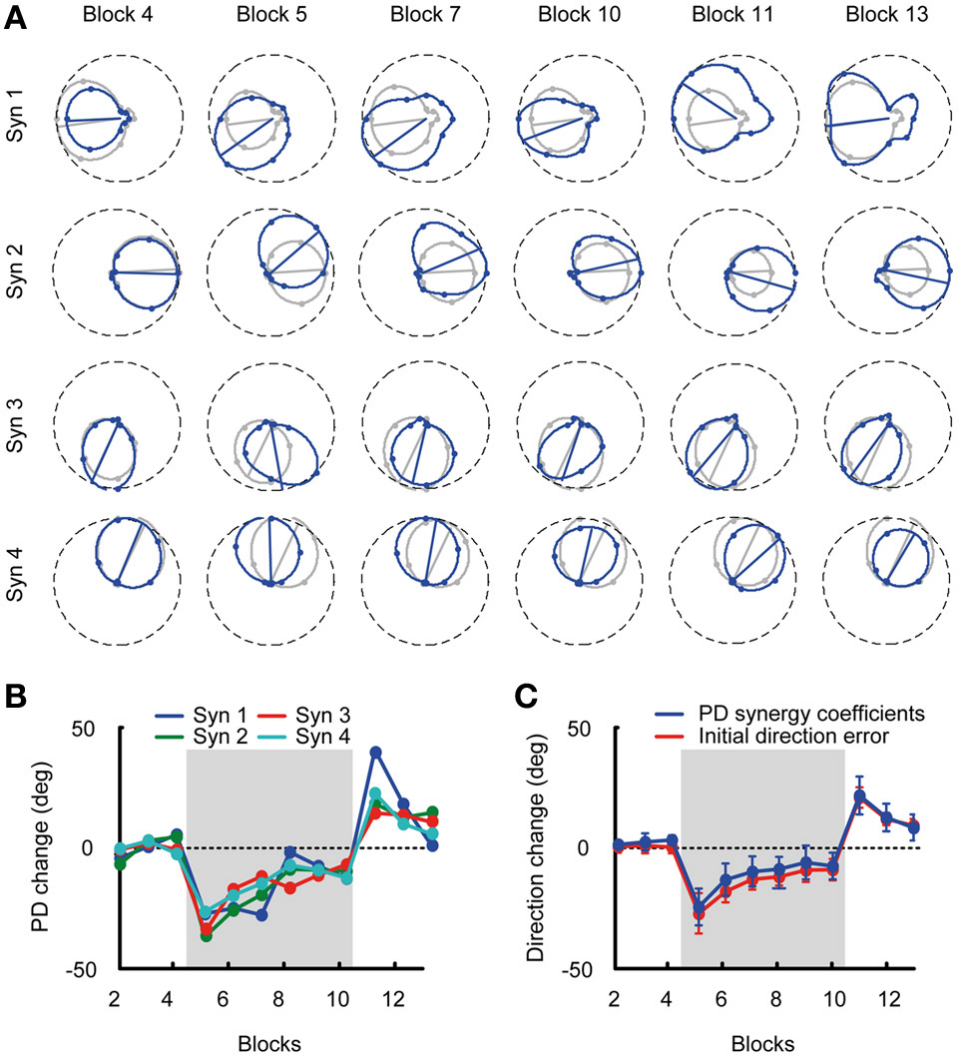
**Tuning curves and preferred directions of synergy coefficients. (A)** Example of tuning curves for the four synergies of subject 3 estimated from different blocks (*blue* markers and lines) compared to the tuning curves calculated from the pooled data of all baseline blocks (reference blocks, *gray* markers and lines). **(B)** Mean PD-change of synergy coefficients for subject 3 for all blocks. **(C)** Grand mean of the PD-change across synergies and subjects (*blue*) and initial direction angle error (*red*) for all blocks. The PD change of the synergy coefficients was not statistically different from the initial direction angle error.

Given the stability of muscle synergies during visuomotor adaptation, we expected the change of PDs of the synergy coefficients to closely match the change of directional error of the initial force and the change of PDs of the muscles. Figure 6A shows an example of the directional tuning and the PDs of the synergy coefficients for subject 3 (*blue tuning curves*) with respect to the tuning and PDs in the reference blocks (*gray*, Blocks 2–4). The change of synergy coefficient PDs from subject 3 is shown across all blocks. The PD change was similar across synergies with −1.43 ± 2.51° (mean ± SD) deviation in the last baseline block (Block 4), an initial deviation of −34.91 ± 0.85° at the beginning of the visuomotor rotation (Block 5), a gradual reduction of PD change (−9.67 ± 4.03°, Block 10), aftereffects at the beginning of the washout phase (19.79 ± 11.43°, Block 11) and a gradual return to baseline (7.52 ± 6.49°, Block 13) at the end of the experiment (Figure 6B). Across subjects the mean PD change of synergy coefficients (Figure 6C, blue traces) was: Block 4: 1.06 ± 4.53, Block 5: −26.95 ± 5.54, Block 10: −9.97 ± 6.02, Block 13: 6.16 ± 7.57, similar to the change of PDs of muscles.

## Discussion

We investigated the changes in the muscle patterns underlying visuomotor adaptation in a virtual reaching task requiring the generation of multidirectional isometric forces with the arm. We found that the changes in the PDs of most of the muscles closely followed the change in force direction required to compensate for the perturbation, suggesting that the adaptive process relies on remapping target directions into new planned force output directions. We then tested whether a given force output is generated, during adaptation to a novel rotation of the force-to-cursor mapping and after re-adaptation to the normal mapping, by the same set of muscle synergies which capture the muscle patterns in the baseline condition. We found that the number and the structure of the synergies was robust throughout adaptation and re-adaptation. In each subject, four or five synergies extracted in the baseline condition could reconstruct with a comparable level of accuracy the muscle patterns recorded before, during, and after the visuomotor rotation and they had a mean similarity with the synergies extracted from individual blocks throughout the experiment significantly higher than the mean similarity between random synergies. We also found that the change in the PDs of the synergy recruitment closely matched the change in the PDs of the individual muscles.

Many studies of motor adaptation after dynamic or visuomotor perturbation have used center-out reaching tasks in which the motion of the hand is either directly perturbed, by viscous force fields generated by a robotic device (Shadmehr and Mussa-Ivaldi, 1994) and by Coriolis forces arising in a rotating room (Lackner and Dizio, 1994), or mapped into a virtual end-effector, as with visuomotor rotation of a cursor on a computer screen (Ghilardi et al., 1995; Imamizu et al., 1995; Krakauer et al., 2000). Only in a few cases isometric force at the hand has been used as motor output instead of hand motion to investigate visuomotor adaptation (Hinder et al., 2007; de Rugy et al., 2009; de Rugy and Carroll, 2010). By using isometric force, as the posture of the arm is fixed, there is no need for visual-proprioceptive recalibration after the perturbation. In contrast, the adaptive response to a visuomotor rotation of the movement of a cursor associated with actual hand movement involves both sensorimotor remapping and sensory recalibration, i.e., alignment of the felt and seen position of the hand at the end of the movement (Simani et al., 2007). Moreover, with isometric force as motor output there is no need for increasing limb impedance by increasing muscle co-contraction to stabilize the hand trajectory immediately after the perturbation, i.e., before it is compensated in the feed-forward motor command. Increase in muscle activation has been reported after dynamic perturbations (Thoroughman and Shadmehr, 1999; Franklin et al., 2003) but also after visuomotor rotation (Paz et al., 2003). In contrast, we did not observe a significant increase in muscle activation, in accordance with a previous study of visuomotor rotation in an isometric reaching task (de Rugy and Carroll, 2010).

Stability of the relationship between directional tuning of the muscles and force has been observed before in macaque monkeys and human subjects after both visuomotor and dynamic perturbations. In monkeys performing a reaching task by moving a joystick that controlled a cursor on a video screen, most muscles recorded in the shoulder, neck and trunk showed clear PDs which were stable in motor coordinates during adaptation to visuomotor rotations (Wise et al., 1998). In humans adapting to viscous force fields, i.e., to velocity dependent forces applied perpendicularly to the hand movement direction, the peak of EMG activity of two pairs of shoulder and elbow muscles counteracting the perturbation gradually shifted earlier in the reaching movement, becoming a feed-forward command, and the EMG tuning curves gradually rotated by an amount specific to the force field (Thoroughman and Shadmehr, 1999). Similarly in monkeys, during adaptation to force fields the PDs of shoulder and elbow muscles rotated in the direction of the external force and returned to baseline when the perturbation was removed (Li et al., 2001), indicating a stable relationship between muscle directional tuning and generated force. In an isometric virtual reaching task in which wrist flexion/extension and radial/ulnar deviation forces generated by human subjects were mapped, respectively, into horizontal and vertical movements of a cursor on a vertically mounted computer screen, the changes in the directional tuning of four wrist muscles closely matched the rotation of the directional error in force after a 45° visuomotor rotation, indicating that the functional contribution of muscles remained consistent during adaptation (de Rugy and Carroll, 2010). Thus, our observations on the stability of the relationship between muscle directional tuning of cosine tuned muscles and force are in accordance with previous observations but they are reported for the first time for adaptation to visuomotor rotation of the isometric force generated by a large number of arm muscles.

Importantly, subjects were not informed on what kind of perturbation (consistent rotation of the force by a fixed angle) they will experience in the experiment. This kind of adaptation is likely to occur implicitly when the desired hand trajectory and the executed trajectory in visual space do not match (Mazzoni and Krakauer, 2006; Krakauer, 2009), possibly by reducing a prediction error computed by a forward model (Shadmehr et al., 2010). A question that arises in this context is why the nervous system does not exploit the redundancy inherent in the neuro-muscular system to compensate the perturbation by reducing the force error at the level of force components generated by individual muscles. Indeed, it would have been possible to compensate for the perturbation by adapting the activation of each muscle reducing the error between force target and muscle force, possibly ending with a muscle pattern in the adapted state different from the muscle pattern selected to generate the rotated force before the perturbation. Despite the theoretical flexibility of the mapping from muscles to forces, i.e., many different muscle patterns can generate the same forces (Kutch and Valero-Cuevas, 2012), adaptation of individual muscles did not appear to be used for compensating the distorted visuomotor mapping. In contrast, the relationship between muscles and forces remained fixed indicating that subjects tried to adapt to the perturbation by rotating the forces (“aiming in a different direction”) from the beginning on. An explanation could be that adaptation to this kind of perturbations occurs early in the sensorimotor transformations mapping visual targets into muscle patterns by adapting a single learning parameter (force direction) resulting in a coordinated rotation of all the muscle PDs. However, adapting only a few high level parameters may be computationally advantageous but it might also be required if the generation of muscle patterns is not as flexible as theoretically possible. The characteristics of the connectivity between different areas in the motor systems might prevent the nervous system from adapting the recruitment of individual muscles to compensate for visuomotor perturbations. Divergence from premotor neurons to many muscles and convergence to a single muscle from many premotor neurons (Graziano, 2006; Rathelot and Strick, 2006) might underlie the organization of muscle synergies in the primary motor cortex (Gentner et al., 2010; Overduin et al., 2012) and in the spinal cord (Hart and Giszter, 2010).

We therefore considered whether the visuomotor adaptation process is compatible with muscle synergies. Muscle synergies has been recently identified during isometric force production (Roh et al., 2012). However, to our knowledge, a direct test of robustness of muscle synergies during visuomotor adaptation has never been conducted. The structure of the synergies and their number appeared to be similar across subjects and blocks. During the adaptation the coefficients of cosine tuned synergies rotated almost identically as the PDs of individual tuned muscles, albeit some synergies contained contributions of non-cosine tuned muscles. Therefore, isometric visuomotor adaptation can be equally well described by rotation of forces, muscle-PD changes, and PD changes of synergy coefficients. Moreover, as adaptation may involve components with different learning rates (Smith et al., 2006) the analysis of muscle synergies may allow to dissociate different components of the adaptive process. A rapidly adapting component may be related to the adjustment of synergy coefficients, e.g., rotating their recruitment to compensate for a visuomotor rotation. A slower component may be involved in the acquisition of new synergies or in changing the structure of existing ones. In isometric visuomotor adaptation we found that only a fast learning component as there is no need for altering the synergies. In contrast, we recently found that adaptation to novel perturbations that cannot be compensated by adapting the recruitment of existing synergies but require new or altered synergies is slower than adaptation to similar perturbations compatible with the synergies (Berger et al., 2013). Testing such perturbations has provided new direct evidence for a synergistic organization (d'Avella and Pai, 2010).

To assess the robustness of the synergies during adaptation we assessed the quality of reconstruction of the muscle patterns recorded throughout the experiment by the synergies extracted from the baseline condition (reference blocks) and their similarity with the same number of synergies extracted from individual blocks. For each subject, the number of synergies was selected as the minimum number sufficient to explain at least 90% of the data variation. While criteria based on a threshold on the variation accounted for (VAF) has been frequently used in the muscle synergy literature (Tresch et al., 1999; Ting and Macpherson, 2005; Torres-Oviedo et al., 2006) other criteria based on the detection of a “knee” in the curve of the VAF as a function of the number of synergies (d'Avella et al., 2003; Cheung et al., 2005; Tresch et al., 2006), on a combination of VAF-threshold and knee (Berger et al., 2013) have been proposed. All these criteria depend on some threshold which must be chosen *ad-hoc*. Recently, a new criterion based on decoding single-trial task parameters from synergy coefficients has been proposed (Delis et al., 2013). Such criterion does not depend on *ad-hoc* parameters but it can be only applied to synergies extracted from a large number of repetitions (>10) of the same experimental condition. Thus, selection criteria for synergies extracted from averaged data or a limited number of repetitions, as in our case, mainly guarantee that the number of synergies can be meaningfully compared across different conditions and subjects rather than ensuring that the “true” number of synergies has been selected. Moreover, to simplify the assessment of synergy similarity we compared the same number of synergies extracted from the reference blocks and extracted from individual blocks. An alternative approach would have been to select a different number of synergies for each block according to the VAF criterion and to assess both the similarity between the pairs formed with the smallest of the two synergy sets and the dimensionality of the set. However, as VAF criterion is affected by noise, we preferred to rely only on the number of synergies selected from three baseline blocks (reference blocks) rather than on the number selected from individual blocks. An incorrect identification of the number of synergies in a single block might in fact significantly affect the mean similarity, as the set with an additional synergy often contains two synergies resulting from the splitting of one of the synergies in the original set (d'Avella et al., 2003) and both synergies can have low similarity with the original one. In any case, the *R*^2^ measure of synergy subspace robustness does not depend on the number of synergies selected for the individual blocks as it is based on the reconstruction of the actual data of each block.

In summary our results indicate that muscle synergy structure is robust during visuomotor adaptation and that the required changes in the muscle patterns are obtained by rotating the directional tuning of the synergy recruitment. Visuomotor adaptation may occur by remapping desired end effector movement into synergy coefficients. Further experiments are required to identify synergies as a physiological correlate of motor learning.

### Conflict of interest statement

The authors declare that the research was conducted in the absence of any commercial or financial relationships that could be construed as a potential conflict of interest.
